# Early-Life Nutrition: The Interplay of Nutritional, Microbial, Genetic, and Environmental Factors in Food Allergy Development and Prevention

**DOI:** 10.3390/nu18183026

**Published:** 2026-09-16

**Authors:** Maria Rogalidou, Tania Siahanidou

**Affiliations:** 1 Division of Gastroenterology, Hepatology & Nutrition, First Department of Pediatrics, Medical School, National & Kapodistrian University of Athens, “Agia Sofia” Children’s Hospital, 11527 Athens, Greece; rogalidoum@yahoo.com; 2 Neonatal Unit, First Department of Pediatrics, Medical School, National & Kapodistrian University of Athens, “Agia Sofia” Children’s Hospital, 11527 Athens, Greece

**Keywords:** food allergy, early-life nutrition, oral tolerance, gut microbiome, complementary feeding, allergenic food introduction, epithelial barrier, immune development, genetic susceptibility, environmental exposures

## Abstract

Background: Food allergy is an important health concern, particularly during infancy and early childhood. Its development reflects complex interactions between genetic susceptibility and early-life nutritional, microbial, epithelial, immunological, and environmental factors. Objective: To examine the role of early-life nutrition in the development and prevention of food allergy, with emphasis on its interplay with the gut microbiome, genetic and epigenetic susceptibility, epithelial barrier function, and environmental exposures. Methods: A structured literature search was conducted primarily in PubMed, supplemented by relevant articles identified through additional bibliographic databases and literature sources. Evidence was thematically synthesized across maternal nutrition, breastfeeding, complementary feeding, allergenic food introduction, dietary diversity, micronutrients, fatty acids, gut microbiome development, genetic and epigenetic mechanisms, and environmental exposures. Results: Early life represents a critical window for immune maturation and the establishment of oral tolerance. Nutritional exposures interact with the gut microbiome, epithelial integrity, and immune pathways, potentially influencing tolerance and sensitization. The most consistent evidence for primary prevention supports the timely introduction and continued ingestion of allergenic foods, particularly peanut and egg, during complementary feeding. Evidence remains insufficient or inconsistent to support maternal allergen avoidance, targeted supplementation, hydrolyzed formulas, probiotics, microbiome-directed interventions, or skin-barrier strategies as established preventive approaches. Associations involving microbiome composition, medication exposure, pollution, and other environmental factors remain heterogeneous and are largely observational. Conclusions: Food allergy reflects complex interactions among diet, microbiota, epithelial barriers, host susceptibility, and environmental exposures rather than a single causal factor.

## 1. Introduction

Food allergy (FA) represents an important public-health burden, particularly in children, although estimated prevalence varies substantially according to the population studied and the methods used for diagnosis and ascertainment. In a US synthesis of 27 surveys involving 452,237 children between 1988 and 2011, self-reported FA increased by approximately 1.2 percentage points per decade. However, heterogeneity in definitions and ascertainment methods prevented estimation of a pooled prevalence, providing stronger evidence for an increase in recognized or reported FA than for biologically confirmed disease [1]. Similarly, longitudinal primary-care data from England showed that the incidence of probable FA doubled between 2008 and 2018, while recorded prevalence increased from 0.4% to 1.1%, reaching 4.0% among children younger than 5 years [2].

European evidence further demonstrates the influence of diagnostic definitions on estimated prevalence. An updated meta-analysis of 110 studies reported a point prevalence of 13.1% for self-reported FA compared with 0.8% when FA was confirmed by oral food challenge, while sensitization was more common [3]. Importantly, sensitization does not necessarily indicate clinical FA. Prevalence is generally highest during infancy and early childhood and may subsequently decline as some childhood food allergies resolve naturally [4]. Beyond prevalence, FA imposes a substantial burden on children and families, healthcare services, and quality of life, including the risk of severe reactions and emergency healthcare utilization [5,6]. These findings underscore the importance of understanding the determinants of FA and identifying effective strategies for prevention, particularly during early life.

Early life represents a critical window for immune development and the establishment of oral tolerance. During pregnancy and infancy, nutritional, microbial, genetic, and environmental signals interact with the developing immune system, influencing epithelial integrity, immune maturation, and gut microbiome development. Following birth, the gastrointestinal tract becomes an important site of immune education, where dietary and microbial antigens are encountered during a period of rapid immune development. The establishment of tolerance to dietary antigens involves coordinated interactions between the intestinal epithelium, immune system, dietary antigens, and gut microbiota, with early-life exposures potentially influencing these processes [7,8,9,10].

FA is increasingly understood as a multifactorial condition arising from interactions between inherited susceptibility and early-life nutritional, microbial, and environmental influences. These factors may act independently or interact across critical developmental periods, shaping immune tolerance and susceptibility to allergic disease. However, evidence regarding their respective contributions remains heterogeneous and, in some areas, inconsistent. Differences in study populations, exposure definitions, timing, and methodological approaches, together with the complex interdependence of early-life factors, make it difficult to disentangle their individual and combined effects on FA risk.

Against this background, early-life nutrition represents a potentially modifiable component of the broader network of factors influencing immune tolerance and FA development. This narrative review aims to synthesize current evidence on the interplay between nutritional, microbial, genetic, and environmental factors during early life and their potential roles in the development and prevention of FA.

## 2. Methods

A structured literature search was conducted primarily in PubMed/MEDLINE from database inception through August 2026. The search strategy used a combination of MeSH terms and free-text terms covering the major topics addressed in this narrative review, including FA, maternal and infant nutrition, breastfeeding, complementary feeding, allergenic food introduction, dietary diversity, micronutrients, fatty acids, infant formula, gut microbiota, microbial metabolites, genetic and epigenetic factors, environmental exposures, and the skin–gut–immune axis. Additional relevant publications were identified through screening the reference lists of key articles and citation tracking. Other bibliographic databases were consulted selectively, when necessary, to identify additional relevant publications; these searches were not conducted systematically or exhaustively. Only English-language publications were considered.

Relevant randomized controlled trials, observational studies, systematic reviews, meta-analyses, guidelines, consensus statements, and selected mechanistic studies were considered when they addressed FA development or prevention in relation to early-life nutrition and interacting biological or environmental factors. Studies unrelated to FA or not addressing the objectives of the review were excluded. Reference lists of relevant publications were also screened to identify additional pertinent studies.

Given the narrative nature of this review, the identified evidence was synthesized qualitatively rather than according to a formal systematic-review or meta-analytic framework. Because this was a narrative review with a non-systematic literature selection process, no predefined number of studies was established. The findings were integrated across nutritional, microbial, genetic, epigenetic, epithelial, environmental, and immunological domains, with emphasis on potential interactions, consistency and strength of the available evidence, and key limitations.

## 3. Development of Oral Tolerance in Early Life

FA develops when immune responses to otherwise harmless food proteins become dysregulated following sensitization. The intestine is a central site for determining whether exposure to dietary antigens results in tolerance or allergic sensitization because it integrates epithelial-barrier function, antigen presentation, microbial signals, and mucosal immune regulation [11,12,13]. The intestinal barrier limits inappropriate penetration of dietary and microbial antigens while allowing controlled antigen sampling and immune education. Disruption of epithelial integrity may increase allergen exposure to underlying immune cells and contribute to sensitization, particularly when combined with genetic susceptibility and an inflammatory mucosal environment [11].

The establishment of oral tolerance during early life depends on coordinated interactions among intestinal epithelial cells, the gut microbiota, antigen-presenting cells, and regulatory T (Treg) cells [13,14]. Following gastrointestinal exposure to dietary antigens, antigen sampling and transport facilitate their presentation by tolerogenic dendritic cells, including within the mesenteric lymph nodes. This process is suggested to promote the differentiation and expansion of antigen-specific Foxp3^+^ Treg cells, which suppress inappropriate immune responses and contribute to the maintenance of intestinal and systemic tolerance [13,14]. Early microbial colonization and dietary diversification may further support the development of microbiota-dependent Foxp3^+^RORγt^+^ Treg populations, which contribute to intestinal immune homeostasis and suppression of type 2 inflammatory responses [13,15].

The gut microbiota contributes to this process through both microbial signals and metabolites. Mechanistic evidence summarized in current reviews indicates that short-chain fatty acids (SCFAs), particularly acetate, propionate, and butyrate, as well as tryptophan-derived metabolites, can influence epithelial integrity, antigen-presenting cells, Treg differentiation, and immune regulation [13,15]. Dietary substrates, particularly fermentable carbohydrates and fiber, can modify microbial metabolism and thereby influence the availability of these immunoregulatory metabolites [16]. Microbial sensing through pattern-recognition receptors, including Toll-like receptors (TLRs), may additionally contribute to the regulation of epithelial and mucosal immune responses [12]. However, the specific contribution of individual microbial taxa or signaling pathways to human FA remains incompletely defined.

When these microbiota–epithelial–immune interactions are disrupted, mechanistic evidence suggests that impaired barrier function or altered regulatory responses may favor type 2 inflammation, allergen-specific IgE production, and sensitization. Conversely, experimental and mechanistic evidence suggests that appropriate gastrointestinal exposure to dietary antigens within a tolerogenic microbial and epithelial environment may promote durable antigen-specific tolerance. Thus, oral tolerance should be viewed as a dynamic process emerging from interactions among dietary antigens, the intestinal barrier, microbiota, microbial metabolites, and regulatory immune pathways during a critical period of immune development [11,12,13,14,15,16].

**Figure 1:**
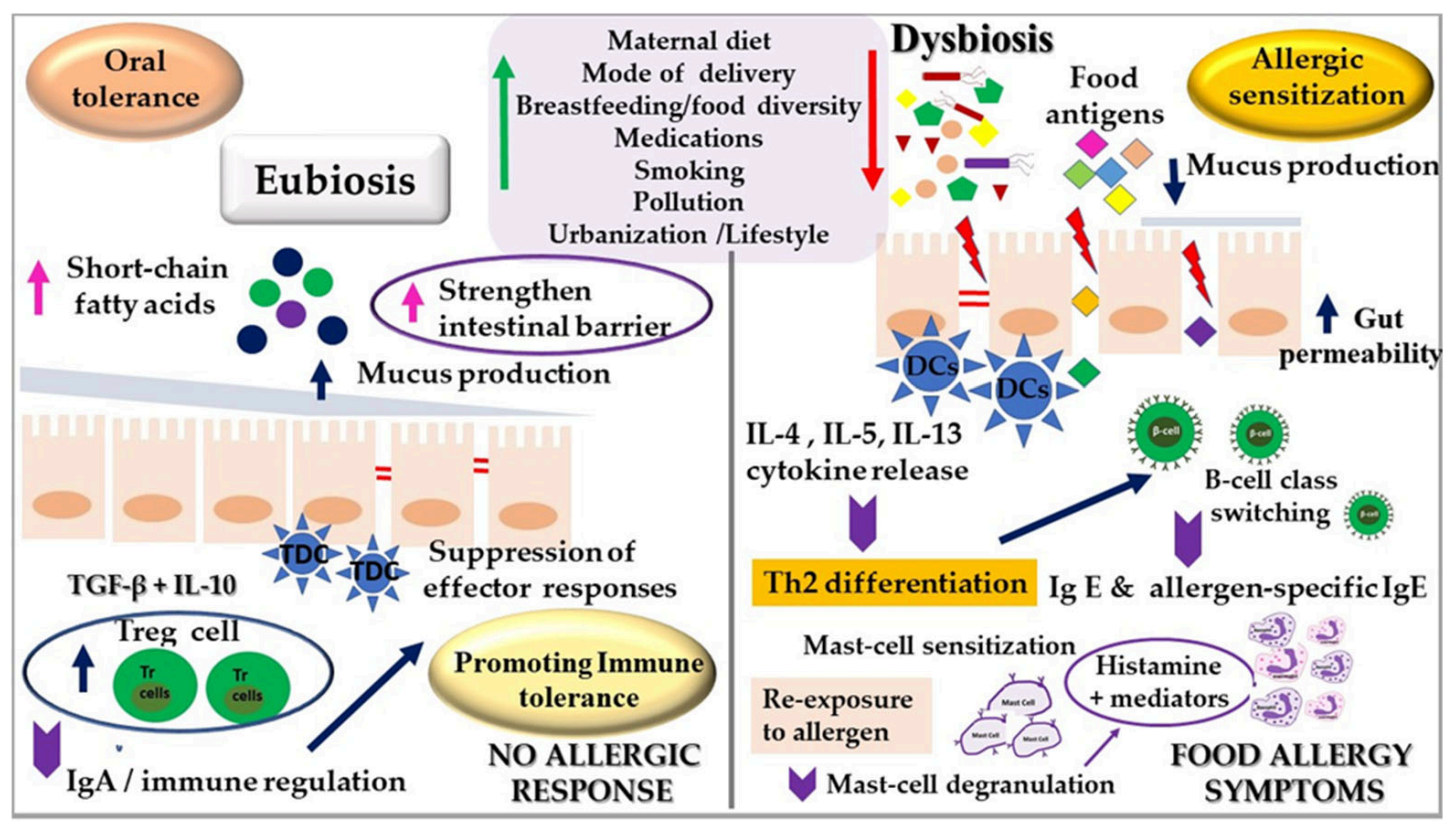
Divergence Between Oral Tolerance and Food-Allergic Sensitization Following Exposure to Early-Life Factors. Early-life exposure to food antigens occurs within a dynamic environment shaped by nutritional, microbial, genetic, and environmental factors. Under homeostatic conditions, gut microbial eubiosis and an intact intestinal epithelial barrier promote tolerogenic antigen presentation by tolerogenic dendritic cells (TDCs), production of regulatory cytokines such as IL-10 and TGF-β, and induction of regulatory T cells (Tregs), thereby supporting oral tolerance and limiting inappropriate immune responses to dietary antigens. In contrast, microbial dysbiosis, intestinal barrier dysfunction, and other susceptibility factors may promote a pro-allergic dendritic-cell phenotype, favoring T-helper 2 (Th2) polarization and production of IL-4, IL-5, and IL-13. These cytokines promote B-cell class switching and the generation of allergen-specific IgE, while IL-5 contributes to eosinophil recruitment and activation. Allergen-specific IgE binds to high-affinity FcεRI receptors on mast cells and basophils, resulting in sensitization. Upon subsequent allergen exposure, cross-linking of receptor-bound IgE triggers mast-cell and basophil degranulation and the release of histamine and other inflammatory mediators. In parallel, eosinophil activation contributes to tissue inflammation and epithelial injury. These processes can lead to the clinical manifestations of FA, including urticaria, gastrointestinal symptoms, respiratory involvement, and, in severe cases, anaphylaxis. Thus, the balance between tolerogenic and allergic immune pathways during early life may represent a critical determinant of subsequent food tolerance or allergy.

As illustrated in Figure 1, early-life nutritional, microbial, genetic, and environmental factors influence the balance between oral tolerance and food-allergic sensitization.

## 4. Early-Life Nutritional Factors and Food Allergy

### 4.1. Maternal Diet and Nutrition During Pregnancy

Maternal nutrition may influence fetal and subsequent infant immune development through effects on nutrient availability, maternal immune responses, placental signaling, and the maternal and infant microbiome. However, current evidence summarized in reviews does not support routine avoidance of common allergenic foods, including peanut, egg, or cow’s milk, during pregnancy as a strategy for preventing childhood FA [10,17,18]. Unnecessary dietary restriction may also compromise maternal nutritional adequacy and overall diet quality.

Several maternal nutritional factors have been investigated in relation to allergic outcomes in offspring, including dietary patterns, vitamin D, omega-3 fatty acids, prebiotics, and probiotics. Although observational and mechanistic studies suggest potential immunomodulatory effects, current evidence from intervention studies remains insufficient to recommend any specific maternal dietary modification for the primary prevention of FA [17,18]. Emerging evidence from systematic reviews and meta-analytic evidence suggests that maternal consumption of certain foods, including eggs, may be associated with a lower risk of eczema or FA in offspring. However, the limited number of randomized studies, heterogeneity in exposure assessment, and potential residual confounding limit the certainty of these findings and prevent firm conclusions [19].

The effects of maternal nutrition are also likely to depend on the broader developmental environment. Maternal nutritional status may interact with the maternal microbiome, placental signaling, fetal immune development, and subsequent infant dietary and environmental exposures. These interactions provide plausible biological pathways linking maternal nutrition with offspring allergy susceptibility, but their clinical relevance in humans remains incompletely defined.

Current evidence therefore favors nutritional adequacy and dietary diversity during pregnancy without unnecessary allergen restriction, rather than targeted maternal dietary interventions for food-allergy prevention [10,17,18,19]. Whether specific dietary patterns or nutrients can modify offspring food-allergy risk remains an important area for prospective and randomized research.

### 4.2. Breastfeeding

Breastfeeding may contribute to the development of oral tolerance through the combined effects of dietary antigens, immunomodulatory factors, and interactions with the infant gut microbiome and intestinal barrier [20,21,22]. Small quantities of maternal dietary antigens can be transferred into breast milk, potentially providing repeated low-dose exposure during a critical period of immune development [20,21]. Human milk also contains a range of bioactive components, including immunoglobulins, cytokines, oligosaccharides, and growth factors, that may influence epithelial integrity, antigen presentation, microbial colonization, and regulatory immune responses [20,22].

Despite these plausible mechanisms, observational evidence regarding whether breastfeeding independently reduces the risk of FA remains inconsistent [7,9,17,20]. Differences in breastfeeding duration and exclusivity, maternal diet, timing of complementary feeding, infant atopic risk, and other socioeconomic and environmental factors complicate interpretation of observational findings. Evidence from randomized studies specifically evaluating breastfeeding as a FA prevention strategy is also limited.

Breastfeeding remains an important component of infant nutrition because of its established nutritional, developmental, and broader health benefits, but its role in preventing FA should not be overstated. Mechanistic evidence suggests that its potential contribution to immune tolerance is more appropriately considered as part of a wider early-life environment that includes intestinal microbial development, skin-barrier integrity, and exposure to dietary allergens during complementary feeding [10,22].

### 4.3. Complementary Feeding, Dietary Diversity, and Introduction of Allergenic Foods

Complementary feeding represents an important developmental period during which dietary antigens are introduced alongside continued maturation of the intestinal barrier, gut microbiome, and mucosal immune system. Current evidence summarized in systematic reviews and guidelines does not support delaying the introduction of complementary or allergenic foods as a strategy for preventing FA, whereas complementary feeding should generally begin when the infant is developmentally ready, typically around 4–6 months of age. Introduction before 4 months specifically for the purpose of food-allergy prevention is not recommended [20,21,22,23,24].

Dietary diversity during complementary feeding may contribute to a favorable nutritional and microbial environment. Observational studies have reported associations between greater dietary diversity and lower risk of allergic disease, but these findings may be influenced by socioeconomic characteristics, feeding practices, healthcare utilization, and other lifestyle factors [25,26]. Consequently, although a varied and nutritionally adequate diet is recommended for healthy infant development, evidence remains insufficient to conclude that dietary diversity itself independently prevents clinically confirmed FA. Thus, dietary diversity should not currently be considered a proven strategy for the primary prevention of FA.

The most consistent evidence concerns the timely introduction of specific allergenic foods, particularly peanut and egg [27,28]. Randomized controlled trials have demonstrated that introduction of peanut during infancy, followed by regular consumption, can substantially reduce the development of peanut allergy, particularly among infants at increased risk [28,29]. In the LEAP trial, regular peanut consumption beginning in infancy markedly reduced peanut allergy at 5 years compared with avoidance among high-risk infants [29]. The preventive effect was accompanied by immunological changes consistent with the development of oral tolerance, including increased peanut-specific IgG4 and reduced peanut-specific IgE responses [29].

Evidence for egg is also supportive, although less consistent than that for peanut. Several randomized trials have suggested that early introduction of well-cooked egg may reduce the risk of egg allergy, with differences between studies related to the age at introduction, preparation method, dose, adherence, and baseline risk [27,28,30,31]. These findings are consistent with the hypothesis that gastrointestinal exposure to allergenic foods during a period of immune maturation may favor antigen-specific tolerance, whereas prolonged avoidance may delay the establishment of tolerance without providing protection against sensitization. The EAT trial evaluated the early introduction of six allergenic foods in 1303 breastfed infants. Although the intention-to-treat analysis did not demonstrate a significant reduction in overall FA, the per-protocol analysis showed lower rates of FA, particularly peanut and egg allergy, among infants who successfully adhered to the intervention [32]. These findings emphasize that successful and sustained consumption may be important when evaluating early allergen introduction and that the preventive effect may vary according to adherence and baseline risk.

Evidence for introducing multiple allergenic foods simultaneously is emerging. In a large cluster-randomized trial, introduction of peanut, cow’s milk, wheat, and egg from approximately 3 months of age reduced FA at 36 months compared with standard care (OR 0.40, 95% CI 0.20–0.80) [33]. However, these findings should be interpreted alongside the stronger and more consistent evidence for peanut and the more heterogeneous evidence for egg and other allergens. Further research is required to determine the optimal timing, quantity, frequency, and duration of exposure to individual and multiple allergens.

Observational studies generally support earlier introduction, although interpretation is complicated by confounding and reverse causation, as families of infants with early allergic symptoms may intentionally delay allergen introduction [34]. Important uncertainties remain regarding the optimal age, quantity, frequency, and duration of exposure, as well as the generalizability of trial findings across populations and risk groups [27,30,34].

Current guidance therefore favors timely introduction of developmentally appropriate allergenic foods during complementary feeding rather than deliberate avoidance or prolonged delay. The European Academy of Allergy and Clinical Immunology (EAACI) guidance supports the introduction of well-cooked egg and age-appropriate peanut during complementary feeding, generally around 4–6 months when developmentally appropriate [23]. More recent guidance similarly emphasizes complementary feeding beginning around this period, dietary diversity, and inclusion of age-appropriate allergenic foods as part of a varied infant diet [35].

The evidence is strongest for peanut, followed by well-cooked egg, whereas evidence for other allergenic foods remains more limited. Importantly, early introduction should be distinguished from a single exposure; available evidence supports continued regular consumption after successful introduction, although the precise optimal dose and frequency remain uncertain [27,28,29,31,32]. In infants with severe atopic dermatitis, established FA, or other features indicating increased risk, introduction of particular allergens may require individualized clinical assessment.

Overall, current evidence supports complementary feeding that is timely, nutritionally adequate, and diverse, with age-appropriate introduction and continued consumption of selected allergenic foods rather than intentional avoidance. Dietary diversity should be viewed as part of a healthy complementary-feeding pattern rather than as an established food-allergy prevention intervention. Complementary feeding should therefore be regarded as one component of a broader prevention strategy involving oral allergen exposure, skin-barrier integrity, microbiome development, and other early-life determinants of immune tolerance.

### 4.4. Vitamin D, Iron, Omega-3 Fatty Acids, Fiber, and Other Dietary Factors

Beyond the timing of allergen exposure, broader dietary patterns and individual nutrients may influence food-allergy development through effects on the gut microbiome, intestinal-barrier integrity, and immune regulation [36,37,38,39]. Current evidence summarized in reviews suggests that diets rich in fiber, fruits, vegetables, and minimally processed foods, including Mediterranean-style dietary patterns, may support microbial diversity and a metabolically favorable intestinal environment. In contrast, dietary patterns characterized by high intakes of saturated fat and refined sugars and low fiber intake have been proposed to promote dysbiosis and inflammatory signaling [36,37,38]. Fiber is of particular interest because its fermentation by gut microorganisms generates metabolites, including short-chain fatty acids, that can influence epithelial integrity and regulatory immune responses. However, evidence linking specific dietary patterns or individual food components to clinically confirmed FA remains largely observational or mechanistic, limiting conclusions about causality and preventing specific dietary patterns from being recommended for food-allergy prevention [36,37,38].

Omega-3 fatty acids have attracted considerable interest because of their potentialanti-inflammatory and immunomodulatory properties. Current evidence summarized in the literature indicates that maternal omega-3 supplementation may influence some allergic outcomes, but the findings have been inconsistent, and evidence for prevention of FA specifically remains insufficient [36]. Vitamin D may also influence epithelial and immune function, with both deficiency and supplementation investigated in relation to allergic disease. However, current evidence does not establish vitamin D supplementation as an effective strategy for preventing FA [37,38,39].

Evidence for iron and other individual micronutrients is similarly insufficient to justify targeted supplementation specifically for food-allergy prevention in infants or pregnant women in the absence of nutritional deficiency [36,37]. The available evidence therefore favors adequate nutritional status and a varied, balanced diet, while the preventive effects of specific nutrients and dietary patterns require confirmation in well-designed prospective and intervention studies [36,37,38,39].

### 4.5. Formula Feeding and the Potential Role of Hydrolyzed Formulas

Hydrolyzed infant formulas have been investigated primarily as a strategy to reduce allergic disease among infants who are not breastfed, particularly those considered to be at increased risk. Earlier studies suggested potential benefits of partially or extensively hydrolyzed formulas, particularly for atopic dermatitis and other allergic outcomes, but evidence for the prevention of FA has remained inconsistent. A recent systematic review and meta-analysis found that extensively hydrolyzed formula may reduce the risk of cow’s-milk allergy during the first two years of life; however, the certainty of evidence was low, and the findings for other allergic outcomes were inconsistent [39]. Similarly, the EAACI concluded that evidence remains insufficient to recommend either for or against partially or extensively hydrolyzed formulas specifically for the primary prevention of FA [23].

Hydrolyzed formulas may therefore have a role in selected clinical circumstances, but their routine use solely for food-allergy prevention in otherwise healthy infants is not supported by current evidence [23,39]. This distinction is important because evidence for effects on atopic dermatitis or other allergic outcomes cannot be directly extrapolated to prevention of clinically confirmed FA. Importantly, evidence suggesting a reduction in atopic dermatitis or other allergic conditions should not be interpreted as evidence of prevention of clinically confirmed FA.

In summary, across early-life nutritional factors, the strength of evidence varies considerably. The most consistent clinical evidence from randomized trials concerns timely introduction and continued consumption of allergenic foods, particularly peanut and egg, whereas evidence for maternal dietary modification, breastfeeding as an independent preventive intervention, dietary diversity, targeted supplementation, and hydrolyzed formulas remains less conclusive [10,17,18,19,20,21,22,23,24,27,28,34,36,37,38,39]. This variation in evidence supports considering early-life nutrition as one component of a broader network involving the gut microbiome, epithelial integrity, immune maturation, genetic susceptibility, and environmental exposures.

The main maternal and infant nutritional factors investigated in relation to food-allergy risk and prevention are summarized in Table 1.

## 5. The Gut Microbiome and Immune Development

The gut microbiome is an important component of immune development, particularly during early life, when the intestinal immune system undergoes rapid maturation. Current evidence summarized in reviews indicates that interactions among intestinal microorganisms, epithelial cells, and immune populations contribute to mucosal homeostasis and the development of tolerance to harmless antigens, including dietary proteins [40]. Mechanistic evidence suggests that microbial signals can influence epithelial function, antigen presentation, Treg-cell development, and inflammatory pathways, providing potential mechanisms through which early microbial exposures may shape susceptibility to FA.

Early-life microbial composition and maturation have been associated with subsequent allergic outcomes. Observational studies of infants who later developed FA have reported differences in microbial diversity, maturation, and the relative abundance of specific bacterial groups, including altered proportions of *Enterobacteriaceae* and *Bacteroidaceae* [41]. Such findings have contributed to the hypothesis that disrupted or delayed microbial maturation may impair the establishment of oral tolerance. However, microbiome findings are heterogeneous across populations and studies, and differences in diet, geography, delivery mode, antibiotic exposure, age, sequencing methods, and allergic phenotype complicate comparisons. Whether specific microbial configurations precede and contribute to FA, or instead reflect other early-life exposures or the disease process itself, remains unresolved.

### 5.1. Microbial Colonization During Pregnancy, Delivery, and Infancy

The infant gut microbiome undergoes substantial establishment and maturation beginning around birth and is shaped by maternal, perinatal, and environmental factors, including delivery mode, feeding practices, antibiotic exposure, and diet. Whether substantial and persistent microbial colonization occurs before birth remains controversial. Although microorganisms have been detected in the placenta, amniotic fluid, and meconium, systematic and narrative reviews have highlighted concerns regarding contamination and methodological limitations that challenge the interpretation of these findings, and a stable, functional prenatal microbiome has not been conclusively demonstrated [42,43,44].

Delivery mode can influence the early trajectory of gut microbial development. A systematic review has reported differences in microbial composition during the first months of life following caesarean section compared with vaginal delivery, including differences in taxa associated with maternal and environmental microbial transmission. These differences tend to diminish with increasing age and are generally less pronounced by approximately six months [45]. Other factors, particularly breastfeeding, complementary feeding, antibiotic exposure, and the household environment, subsequently contribute to microbial maturation.

Mechanistic and observational evidence suggests that early microbial development may be relevant to immune maturation because microbial signals interact with the intestinal epithelium and mucosal immune system during a period of substantial developmental plasticity. Microbial products can influence epithelial integrity, antigen presentation, regulatory immune pathways, and the differentiation of immune cell populations involved in oral tolerance [44]. Perturbations in these processes may modify the intestinal environment in which tolerance to dietary antigens is established. However, evidence linking specific perinatal microbial exposures directly to subsequent FA remains limited and difficult to interpret, because these exposures frequently cluster with maternal characteristics, feeding practices, medication use, and other environmental factors [42,43,44,45].

### 5.2. Perinatal Medical Exposures: Effects of Caesarean Delivery, Antibiotics, Acid-Suppressive Medications, and Feeding Practices

Early-life medical and feeding practices may influence the developmental trajectory of FA through effects on the gut microbiome, intestinal-barrier function, dietary antigen exposure, and immune maturation. Caesarean delivery and exposure to microbiome-modifying medications, particularly antibiotics and acid-suppressive medications (ASMs), have received considerable attention. However, associations with FA remain difficult to distinguish from underlying maternal, perinatal, clinical, and socioeconomic characteristics.

Caesarean delivery has been associated with a modestly increased risk of FA. A 2024 systematic review and meta-analysis including 113 studies reported an association between caesarean delivery and FA (OR 1.35, 95% CI 1.18–1.54) [46]. Altered microbial exposure during birth has been proposed as one potential mechanistic explanation, as caesarean delivery can modify the early acquisition and composition of the infant gut microbiome. However, systematic review evidence indicates that many microbiome differences associated with delivery mode are relatively transient, and their long-term biological significance remains uncertain [45]. Furthermore, residual confounding related to maternal characteristics, the indication for caesarean delivery, perinatal complications, antibiotic exposure, breastfeeding, and other feeding practices limits causal interpretation [46]. Caesarean delivery should therefore be considered a potential modifier or marker of the early-life microbial environment rather than an established independent cause of FA.

Antibiotic and ASM exposure have similarly been investigated as potential modifiers of FA risk because experimental and mechanistic evidence indicates that both can alter microbial ecology, gastrointestinal antigen exposure, epithelial-barrier function, and immune development. Antibiotic exposure has been associated with subsequent food-allergic outcomes in several large observational studies. A systematic review and meta-analysis involving more than 21 million children reported an association between prenatal antibiotic exposure and FA (OR 1.25, 95% CI 1.09–1.44) [47]. A large US Medicaid population-based cohort study found that, antibiotic exposure during early childhood was associated with an increased risk of FA (HR 1.40, 95% CI 1.34–1.45) [48], while a meta-analysis of observational studies reported an association between early-life antibiotic exposure and FA (OR 1.42, 95% CI 1.08–1.87), although no significant association was observed with objective markers of atopy, including skin-prick-test positivity and allergen-specific IgE [49]. Prenatal and early postnatal antibiotic exposure have also been associated with specific food-allergic outcomes, including cow’s-milk allergy [50], although substantial heterogeneity exists with respect to exposure timing, indication, duration, antibiotic class, and cumulative exposure [47,51,52].

Acid-suppressive medications, particularly proton-pump inhibitors (PPIs), have received similar attention because mechanistic evidence suggests that suppression of gastric acidity may alter dietary-protein digestion, the form and quantity of dietary antigens reaching the intestine, and gastrointestinal microbial ecology. Maternal acid-suppressive medication use during pregnancy was associated with increased odds of FA at 12 months in a prospective observational cohort study (adjusted OR 2.33, 95% CI 1.07–5.07) [53], while a systematic review and meta-analysis reported an association between PPI exposure and childhood FA (OR 2.65, 95% CI 1.22–5.77), with no statistically significant association for H2-receptor antagonists [51].

Biologically, experimental and mechanistic evidence suggests that antibiotic- and ASM-related alterations in microbial composition, microbial metabolites, epithelial-barrier function, and regulatory immune pathways could plausibly influence the establishment of oral tolerance. Nevertheless, the predominantly observational nature of the evidence limits causal inference. Confounding by indication is particularly important because infants and pregnant women receiving these medications may differ from unexposed individuals in infection burden, gastrointestinal symptoms, underlying disease, hospitalization, healthcare utilization, concomitant medication exposure, and other characteristics associated with allergic susceptibility.

Differences in timing, duration, and underlying health may also influence the magnitude and persistence of any microbiome effects [42,43,44,45,46,47,51,54]. Thus, antibiotics and ASMs are best regarded as potential modifiers or markers of early-life susceptibility rather than established direct causes of FA. From a prevention perspective, these findings support appropriate rather than indiscriminate avoidance of medical exposures: caesarean delivery should be determined by obstetric indications, while antibiotics and ASMs should be used when clinically indicated, with unnecessary or prolonged exposure avoided. Current evidence does not justify modifying medically indicated delivery or withholding necessary medications specifically to prevent FA.

Feeding practices including breastfeeding and complementary feeding provide an additional influence on microbial and immune development.

Maternal diet may also shape the maternal and infant microbial environment. In a sub-cohort of 430 mother–infant pairs from the MEDALLION study, an observational cohort analysis found that greater adherence to a Mediterranean dietary pattern during pregnancy and lactation was associated with slightly lower odds of FA in offspring (adjusted OR 0.94; 95% CI 0.89–1.00 for pregnancy and 0.88–1.00 for lactation) [55]. Given that the confidence intervals reached 1.00, these associations should be interpreted cautiously.

Associations were also reported for individual dietary components, including fruits, vegetables, full-fat dairy, meat, and fish [55]. However, these findings are observational and may reflect broader dietary and lifestyle patterns rather than independent effects of individual foods. They therefore provide useful hypotheses regarding the maternal diet–microbiome–immune axis but do not establish specific foods as preventive or harmful exposures.

### 5.3. Microbial Metabolites, Especially SCFAs

Microbial metabolites provide an important link between dietary intake, gut microbiota, intestinal barrier function, and immune regulation. SCFAs, particularly acetate, propionate, and butyrate, are produced through microbial fermentation of dietary fiber and can influence host physiology through G-protein-coupled receptors and histone deacetylase inhibition. Experimental and mechanistic studies suggest that these pathways may support epithelial barrier integrity, modulate inflammatory signaling, and promote regulatory immune responses involved in oral tolerance [56,57].

Early-life SCFA profiles have been associated with allergic outcomes. A systematic review of 37 studies found that higher concentrations of major SCFAs during early life were generally associated with a lower risk of allergic diseases, including IgE-mediated FA, although associations varied according to the metabolite, outcome, and age at assessment [57]. These findings support an observational association between dietary fiber, microbial metabolism, and immune regulation. However, evidence that increasing SCFA production through dietary or microbiome-directed interventions can directly prevent FA remains limited.

Beyond SCFAs, the gut microbiome produces diverse metabolites, including branched-chain fatty acids, tryptophan- and tyrosine-derived compounds, secondary bile acids, sphingolipids, histamine, and polyamines, which experimental and mechanistic studies suggest may influence epithelial integrity and immune responses [58]. These metabolites can affect regulatory T-cell and Th17 pathways as well as inflammatory signaling, suggesting that microbial metabolic activity may be relevant to the development of immune tolerance [58]. Accordingly, investigation of the microbiome–metabolite axis may complement assessment of microbial composition and provide additional insight into FA development than assessment of microbial composition alone. Longitudinal studies are needed to determine which metabolic changes precede the development of FA and whether they represent causal pathways or potential targets for prevention.

### 5.4. Gut Barrier Integrity and Oral Tolerance

The intestinal barrier is a dynamic interface comprising the gut microbiota, mucus layer, intestinal epithelium, and mucosal immune system. These components regulate interactions between luminal contents and host tissues while maintaining intestinal homeostasis [59,60]. During early life, the barrier undergoes substantial maturation and is continuously exposed to new dietary and microbial stimuli. Review and mechanistic evidence suggest that diet, infections, antibiotic exposure, and other environmental factors may influence epithelial integrity and intestinal permeability, thereby modifying the extent and context of antigen exposure [59,60].

Intestinal permeability is particularly relevant to FA development because epithelial integrity determines the access of dietary and microbial antigens to underlying immune compartments. Experimental and mechanistic studies suggest that disruption of the epithelial barrier may increase antigen penetration and facilitate interactions with immune cells, particularly in the presence of genetic susceptibility or local inflammatory signals. Conversely, preservation of barrier integrity may restrict inappropriate antigen exposure while permitting regulated antigen sampling and mucosal immune education. The broader mechanisms through which dietary antigen exposure promotes oral tolerance are discussed in Section 3.

Early-life microbial development may contribute to barrier maturation through interactions with epithelial cells and regulation of the mucosal environment. Observational studies have reported alterations in microbiome development in children who subsequently develop FA, although the direction and clinical significance of these associations remain uncertain [7,60]. Microbial metabolites may also influence epithelial function; however, mechanistic evidence suggests that their contribution should be considered within the broader context of diet, host factors, and immune development rather than as independent determinants of barrier integrity [56,58].

Nutritional factors may further modify intestinal barrier function. Dietary components, including fatty acids, carbohydrates, proteins, vitamins, and probiotics, have been investigated for their potential effects on epithelial integrity and immune regulation [16]. Nevertheless, evidence from intervention and observational studies remains inconsistent, and no single dietary or microbial intervention has been established as sufficient to prevent FA.

Overall, current evidence supports intestinal barrier integrity as one component of a multifactorial system linking early-life environmental exposures with FA risk. Mechanistic evidence provides biological plausibility, while human observational evidence remains heterogeneous regarding the extent to which barrier dysfunction contributes causally to FA. Interactions among epithelial function, microbial development, dietary exposure, and host immune responses are likely to influence whether encounters with dietary antigens occur in a tolerogenic or sensitizing context.

### 5.5. Dysbiosis and Associations with Food-Allergic Phenotypes

The first 1000 days of life represent a critical period for the establishment and maturation of the gut microbiome, during which maternal microbial transmission, diet, environmental exposures, and immune development interact. Observational and review evidence indicates that alterations in early-life microbial composition and function are associated with subsequent food-allergic phenotypes [7,61,62]. However, these associations are heterogeneous, and their causal significance remains uncertain.

A scoping review of 34 studies identified differences in gut microbial composition between children with FA and healthy controls, including a greater abundance of *Enterobacteriaceae*, *Clostridium sensu stricto*, *Ruminococcusgnavus*, and *Blautia*, together with lower abundance of *Bifidobacteriaceae*, *Lactobacillaceae*, and certain *Bacteroides* species [61]. These findings were not consistent across all the studies. Differences in age, diet, geographical location, antibiotic exposure, feeding practices, study design, and allergic phenotype may contribute to the observed variability [61,63]. Accordingly, current evidence does not support a single, reproducible microbial signature of FA. Importantly, a systematic review of children with cow’s-milk allergy identified dysbiosis that may involve changes in microbial function as well as taxonomic composition. In children with cow’s-milk allergy, systematic evidence has identified alterations in microbial communities together with changes in microbial metabolites, including SCFAs and amino-acid metabolism [64]. Systematic reviews have likewise reported associations between SCFA profiles and allergic outcomes, although substantial heterogeneity exists among studies and the direction of these associations is not uniform [56,57]. These observations suggest that microbial metabolic activity and host–microbe interactions may provide information that is not captured by measures of microbial diversity or individual bacterial taxa alone.

Microbiome alterations may also differ according to allergic phenotype. In children with early-onset atopic dermatitis and FA, an observational study reported distinct microbial and metabolic profiles, including alterations in methionine metabolism and PPAR-γ-related pathways [65]. Similarly, differences in specific bacterial genera have been observed in young children with FA despite the absence of significant differences in overall microbial diversity [66]. Such findings support the possibility that microbiome–host interactions may vary according to disease phenotype and developmental stage.

Several factors complicate interpretation of the available evidence. Review and observational evidence indicate that microbiome composition is strongly influenced by diet, geography, antibiotic exposure, delivery and feeding practices, age, and other environmental factors [42,43,44,45,46,47,48,49,61,63]. In addition, dietary restriction following diagnosis may itself alter microbial composition and metabolic activity, making it difficult to determine whether dysbiosis is a cause, consequence, or correlate of FA. Longitudinal studies beginning before the development of allergic disease are therefore particularly important for establishing temporal relationships.

Overall, current evidence from observational studies and systematic/scoping reviews supports an association between early-life alterations in the gut microbiome, microbial metabolism, and food-allergic phenotypes, but causality has not been established [7,61,62,64,66]. Future studies integrating longitudinal microbiome profiles with microbial metabolites, dietary exposures, epithelial function, immune responses, and well-defined clinical phenotypes may help determine whether microbiome-related markers can contribute to the prediction, prevention, or treatment of FA.

## 6. Genetic and Epigenetic Determinants

Genetic and epigenetic factors may contribute to FA susceptibility through effects on epithelial-barrier function, antigen presentation, and immune regulation. These factors are considered in this review because they may modify how early-life nutritional and microbial exposures influence immune development and the balance between tolerance and allergic sensitization. Current evidence supports a multifactorial model in which inherited susceptibility interacts with early-life nutrition, environmental exposures, microbiome development, and immune maturation [67].

### 6.1. Genetic Susceptibility and Family History

Genetic susceptibility contributes to FA, although FA is a complex polygenic disorder rather than a condition determined by a single gene. Family-based and genetic association studies support a heritable component, while genome-wide association studies have identified multiple loci associated with FA susceptibility, including variants in or near genes involved in epithelial-barrier function and immune regulation, such as *FLG*, *SERPINB7*, *HLA*, and *IL4* [67]. However, the contribution of individual variants appears to vary across populations and allergic phenotypes, and no single genetic marker currently provides sufficient predictive value for clinical use.

Evidence summarized in a systematic review and meta-analysis of 190 studies involving approximately 2.8 million participants indicates that parental and sibling allergic disease are associated with increased risk of childhood FA. The association was particularly evident when FA was present in a first-degree relative, with increased odds among children with an affected mother (OR 1.98), father (OR 1.69), both parents (OR 2.07), or sibling (OR 2.36) [52]. Similarly, the population-based HealthNuts study of 5276 one-year-old infants, including 534 children with oral food challenge-confirmed FA, reported a modest increase in risk among infants with one immediate family member with allergic disease (OR 1.4, 95% CI 1.1–1.7). Risk was higher among infants with two or more affected family members (OR 1.8, 95% CI 1.5–2.3), with some variation according to the specific allergen, including egg and peanut [68].

These findings indicate that familial allergic history may help identify children at increased risk, although familial aggregation may reflect both shared genetic susceptibility and common environmental exposures. Genetic predisposition alone does not determine whether FA develops. Instead, inherited susceptibility may interact with early-life factors such as epithelial-barrier integrity, microbiome development, antibiotic exposure, dietary exposures, and the timing and route of allergen exposure [52,67,68].

FA is therefore best understood within a gene–environment interaction model, in which genetic variation modifies susceptibility while nutrition, environmental exposures and immune maturation influence the development of tolerance or allergic sensitization.

### 6.2. Epithelial-Barrier Genes and Immune-Regulatory Pathways

Genetic associations with FA particularly involve pathways related to epithelial-barrier integrity and immune regulation. Variants in *FLG* and *SERPINB7* have been investigated in relation to epithelial-barrier function, whereas the *HLA* region and *IL4* are involved in antigen presentation and type 2 immune responses [67,68,69]. *FLG* loss-of-function variants are strongly associated with impaired skin-barrier function and atopic dermatitis and may indirectly increase susceptibility to food sensitization by facilitating allergen penetration through the skin. Associations involving the HLA region, including those reported for peanut allergy, further suggest that genetic variation in antigen presentation may influence allergen-specific immune responses [70].

The relationship between barrier integrity and allergen exposure is particularly relevant in children with atopic dermatitis. Disruption of the skin barrier may facilitate penetration of environmental and food allergens and promote allergen-specific IgE responses. This concept is consistent with the dual-allergen-exposure hypothesis, whereby exposure to allergens through an inflamed or disrupted skin barrier may promote sensitization, whereas gastrointestinal exposure may favor oral tolerance [71]. Thus, the route, timing, and context of allergen exposure may interact with host susceptibility and early-life feeding practices to influence the development of allergy.

Genetic variants are therefore more likely to modify susceptibility than independently determine the development of FA. Their effects may be influenced by epithelial-barrier integrity, immune maturation, microbiome development, dietary exposures and early-life environmental factors [67,70,71,72]. This supports a multifactorial model in which genetic predisposition with nutrition and other early-life exposures interacts to influence whether exposure to dietary antigens results in tolerance or allergic sensitization.

### 6.3. Gene–Environment Interactions: Diet and Microbiome

The available evidence indicates potential interactions among diet, the gut microbiome, intestinal-barrier function, and immune regulation in FA, but remains insufficient to define specific gene–diet or gene–microbiome interactions. Early-life microbial exposures, dysbiosis, dietary factors, and microbial metabolites have been associated with FA susceptibility and mechanisms involved in oral tolerance, including regulation of epithelial-barrier integrity, type 2 immune responses, and regulatory T-cell development [73,74,75,76]. Dietary components such as fiber, tryptophan, unsaturated fatty acids, polyphenols, vitamins, and probiotics may influence these pathways, whereas high-fat dietary patterns may promote microbial alterations and inflammatory signaling [74,75,76].

Mechanistic evidence is derived predominantly from experimental models and mechanistic studies, whereas human evidence remains largely observational. Clinical evidence for microbiome-directed interventions, including probiotics, prebiotics, symbiotics, and fecal microbiota transplantation, remains limited and heterogeneous [73,74,75,76]. Furthermore, current studies have not established specific host genetic variants that consistently modify responses to dietary or microbial exposures. Quantified genotype-by-diet and genotype-by-microbiome effects are also largely unavailable. Gene–diet and gene–microbiome interactions should therefore be considered plausible biological mechanisms rather than established determinants of food-allergy risk [67,70,72,73,74,75,76].

Future research combining genomic, epigenomic, dietary, microbiome, metabolomic, and immune data may clarify these interactions and determine whether they can improve individual risk prediction or contribute to personalized strategies for food-allergy prevention.

### 6.4. Epigenetic Programming During the Prenatal and Early-Life Period

Epigenetic mechanisms may contribute to food-allergy susceptibility by regulating gene expression during critical periods of immune and epithelial development. DNA methylation, histone modifications, and non-coding RNAs can be influenced by maternal nutrition, environmental exposures, inflammation, and early-life microbial signals, potentially affecting pathways involved in epithelial-barrier integrity and immune tolerance [77,78,79].

Observational studies have identified differences in epigenetic profiles associated with allergic phenotypes, suggesting that epigenetic regulation may contribute to the development or persistence of FA [77,78,79,80]. However, the findings remain heterogeneous, and the direction of these associations is uncertain. Epigenetic alterations may precede disease and contribute to susceptibility, arise as a consequence of allergic inflammation, or reflect exposure to environmental factors. Consistent with this uncertainty, a study from the CHILD cohort found no significant association between DNA methylation at age one and either sensitization to or introduction of highly allergenic foods, highlighting the inconsistency of current evidence [81].

Mechanistic evidence suggests that epigenetic mechanisms may provide a biological link between genetic susceptibility and prenatal or early-life environmental exposures. Maternal nutrition, specific dietary components, environmental factors, and microbiome-derived metabolites may influence epigenetic regulation during fetal and infant immune development [78,79]. This possibility has generated interest in nutritional and environmental approaches capable of modifying epigenetic pathways; however, evidence that such interventions can prevent FA remains insufficient [78,82].

Epigenetic regulation is therefore best considered a potential mediator of gene–environment interactions rather than an established independent cause of FA. Specific epigenetic signatures may eventually have value as biomarkers for food-allergy susceptibility, persistence, resolution, or treatment response, but their clinical utility has not been established [77,79,80]. Longitudinal studies incorporating repeated epigenetic measurements with dietary, microbiome, environmental, and clinical data are needed to clarify temporal relationships and determine whether epigenetic changes are causally involved and potentially modifiable [77,80,82].

## 7. Environmental Influences

Environmental and metabolic factors may influence food-allergy susceptibility partly by modifying the effects of early-life nutrition and microbiome development. In observational studies, FA and the increasing prevalence of FA have been associated with changes in dietary patterns, antibiotic exposure, urbanization, environmental pollutants, and alterations in the gut microbiome. The intestinal epithelial barrier is an important interface linking dietary antigens, microorganisms, and immune cells. Its integrity may be influenced by genetic susceptibility, diet, microbial signals, inflammation, and other environmental exposures. Mechanistic evidence suggests that disruption of barrier function may increase exposure of the immune system to dietary and microbial antigens and contribute to allergic sensitization [12]. These interactions highlight the environment as a context in which nutritional, microbial, genetic, and immunological factors converge.

### 7.1. Pollution and Tobacco Smoke

Early-life exposure to air pollution and other environmental pollutants may influence food-allergy susceptibility through effects on epithelial-barrier integrity, oxidative stress, and immune regulation, although evidence for clinically confirmed FA remains inconsistent [83,84,85,86,87,88]. A recent systematic review and meta-analysis of 21 observational studies involving 120,454 participants found that exposure to PM_2.5_ was associated with increased odds of childhood FA (OR 1.20, 95% CI 1.01–1.42), whereas no significant associations were observed for PM_10_, NO_2_, or SO_2_. Exposure to mold or dampness was also associated with increased odds of FA (OR 1.55, 95% CI 1.23–1.95) [83]. Prenatal exposure to PM_2.5_ and NO_2_ has also been associated with childhood allergic diseases in observational studies, although evidence specifically regarding FA remains limited [84].

Evidence concerning tobacco smoke is less consistent. A systematic review and meta-analysis of 32 observational studies involving approximately 190,000 children found no consistent association between parental smoking and FA or food sensitization during early childhood [85]. Nevertheless, mechanistic evidence suggests that tobacco smoke may affect epithelial and immune pathways through oxidative and inflammatory mechanisms. Other environmental exposures, including traffic-related pollutants, pesticides, heavy metals, endocrine-disrupting chemicals, and alterations in the microbial environment, have also been proposed as potential contributors to food-allergy susceptibility [86,87,88].

The heterogeneity of current findings may reflect differences in exposure assessment, timing and duration of exposure, geographical variation, genetic background, and co-exposure to multiple environmental factors [86,87,88]. Importantly, much of the available evidence is observational, limiting conclusions regarding causality. Environmental exposures may therefore act as modifiers of food-allergy susceptibility rather than independent causes, potentially interacting with nutrition, genetic predisposition, microbiome development, epithelial integrity, and immune maturation [83,84,85,86,87,88]. Further prospective studies using objective exposure measurements and clinically confirmed food-allergy outcomes are needed to clarify these relationships and identify potentially modifiable environmental targets for prevention.

### 7.2. Urbanization and Lifestyle

Urbanization and modernization have been proposed as contributors to the increasing prevalence of FA, although the underlying mechanisms remain uncertain. Observational studies have reported higher prevalence of FA in highly urbanized and Westernized populations than in some rural settings, suggesting that differences in environmental and lifestyle exposures may influence susceptibility [84,85,86]. Proposed factors include Westernized dietary patterns, reduced dietary fiber intake, greater consumption of processed foods, increased antibiotic and pollutant exposure, reduced contact with natural environments and biodiversity, and changes in the gut microbiome [87,88,89,90,91].

Dietary changes may be particularly relevant within this broader environmental transition. Westernized dietary patterns may affect FA susceptibility through alterations in microbial composition, intestinal-barrier integrity, and immune regulation. However, dietary patterns are closely linked to other features of modern lifestyles, making it difficult to determine the independent contribution of individual factors [89,92]. Similarly, the hygiene, “old friends,” biodiversity, and dysbiosis hypotheses suggest that reduced exposure to diverse microorganisms during early life may influence immune maturation and allergic susceptibility [90,91]. Although these concepts provide plausible biological mechanisms, direct evidence linking reduced microbial exposure specifically to clinical FA remains limited.

Socioeconomic and lifestyle factors may further modify these relationships. Differences in dietary quality, healthcare access, medication exposure, and living conditions may contribute to variation in FA risk and management [92,93]. Food insecurity is particularly relevant because limited access to nutritious foods may influence dietary quality and the ability of families to manage FA safely [93].

Urbanization should therefore be viewed as a contextual determinant rather than a direct cause of FA. Its potential effects likely arise from the combined influence of diet, microbiome development, environmental exposures, and immune maturation [89,90,91,92,93]. Future longitudinal studies should aim to distinguish the individual and combined contributions of these factors and identify modifiable components of the modern early-life environment.

### 7.3. Pets, Farm Environments, Siblings, and Microbial Diversity

Early-life exposure to farm environments, pets, and older siblings may influence allergic disease through increased microbial exposure and effects on immune maturation. Evidence for protective associations is stronger for atopy and allergic sensitization than for clinically confirmed FA [94,95].

A systematic review reported that early-life exposure to farm environments was generally associated with a lower risk of atopy in school-aged children, although differences in exposure definitions and outcome assessment limited comparisons across studies [88]. Similarly, a meta-analysis of 114 studies found that being second-born or later was associated with a modestly lower risk of FA (RR 0.77, 95% CI 0.66–0.90) [94]. In a prospective birth cohort study, greater sibling exposure was associated with more mature gut microbiota at 12 months, and greater microbiota maturation was subsequently associated with lower odds of FA (OR 0.45, 95% CI 0.33–0.61) [96]. These findings suggest a potential link between microbial exposure, microbiome development, and allergic susceptibility, although causality cannot be established.

Farm residence and pet exposure have also been associated with differences in infant gut microbial composition, with some microbial patterns linked to lower subsequent allergic outcomes [97]. However, the specific microorganisms or microbial functions responsible for these associations remain uncertain. Differences in household characteristics, diet, socioeconomic factors, environmental exposures, and parental allergic status may also contribute to the observed relationships [62,96,97].

Taken together, early-life exposure to diverse microbial environments may contribute to immune maturation and tolerance. However, current evidence does not establish farm living, pet ownership, or sibling exposure as specific preventive interventions for FA. These exposures may be relevant primarily as components of the broader microbial environment interacting with early-life nutrition and microbiome development [62,96,97].

### 7.4. Daycare and Food Allergy

Daycare attendance has been investigated as a potential modifier of FA risk because early childcare may influence exposure to microorganisms, infections, and dietary allergens. However, available evidence remains inconsistent and does not establish daycare attendance as either a risk or protective factor for FA [88,98].

In a Swedish study of 10,851 children, daycare attendance was associated with a higher frequency of reported food-related allergic reactions, particularly among children aged 1–4 years [98]. In contrast, other studies have reported no association with atopic disease or a potentially lower risk of challenge-confirmed FA among children attending daycare during early infancy [88]. Differences in the age at daycare entry, duration of attendance, microbial exposures, feeding practices, and household characteristics may partly explain these discrepant findings.

Host characteristics may further influence responses to daycare-related microbial exposure. A prospective analysis of two birth cohorts reported that associations between early daycare attendance and sensitization or atopic wheezing varied according to TLR2 genotype, suggesting that genetic variation may modify immune responses to microbial exposures [99]. However, these findings concern allergic phenotypes more broadly and cannot be directly extrapolated to FA.

Daycare attendance is therefore best regarded as a marker of the broader early-life microbial and social environment, rather than an established independent determinant of FA. Current observational evidence remains insufficient to support recommendations regarding daycare attendance for FA prevention [88,98,99,100].

### 7.5. Vaccinations and Food Allergy Risk

Routine childhood vaccination has not been established as a risk factor for FA. Systematic reviews have found no evidence that routine infant vaccination increases the risk of allergic disease or FA [101,102]. Although BCG vaccination has been hypothesized to influence immune development, evidence has not demonstrated consistent protection against FA [88,102].

Current evidence therefore does not support childhood vaccination as a meaningful determinant of food-allergy development or as a preventive intervention. Vaccination should continue to be guided by established public-health recommendations rather than considerations related to food-allergy risk [101,102,103].

### 7.6. Obesity and Food Allergy Risk

Obesity may influence food-allergy susceptibility through chronic low-grade inflammation, immune dysregulation, and alterations in intestinal-barrier function. Adipose tissue produces inflammatory mediators and adipokines, including leptin, which can affect lymphocyte activity, cytokine production, and regulatory T-cell responses. These changes may create a pro-inflammatory environment that favors allergic immune responses [104,105].

In a population-based study of U.S. children and adolescents, obesity was associated with higher total IgE concentrations and increased odds of food sensitization (OR 1.59; 95% CI, 1.28–1.98). The association with food sensitization was stronger than that observed for overall atopy, while C-reactive protein was also associated with IgE levels and sensitization, supporting a possible contribution of systemic inflammation [106]. However, sensitization does not necessarily indicate clinical FA, and evidence directly linking childhood obesity with clinically confirmed FA remains limited.

Experimental evidence provides additional mechanistic support. In animal models, high-fat diet-induced obesity has been associated with increased intestinal permeability, mast-cell activation, enhanced Th2 responses, and more severe food-allergic reactions [107]. High-fat diets may also alter the gut microbiome, and transfer of obesity-associated microbiota increased susceptibility to FA in germ-free mice, suggesting a potential role for microbiome-mediated pathways [108].

**Figure 2:**
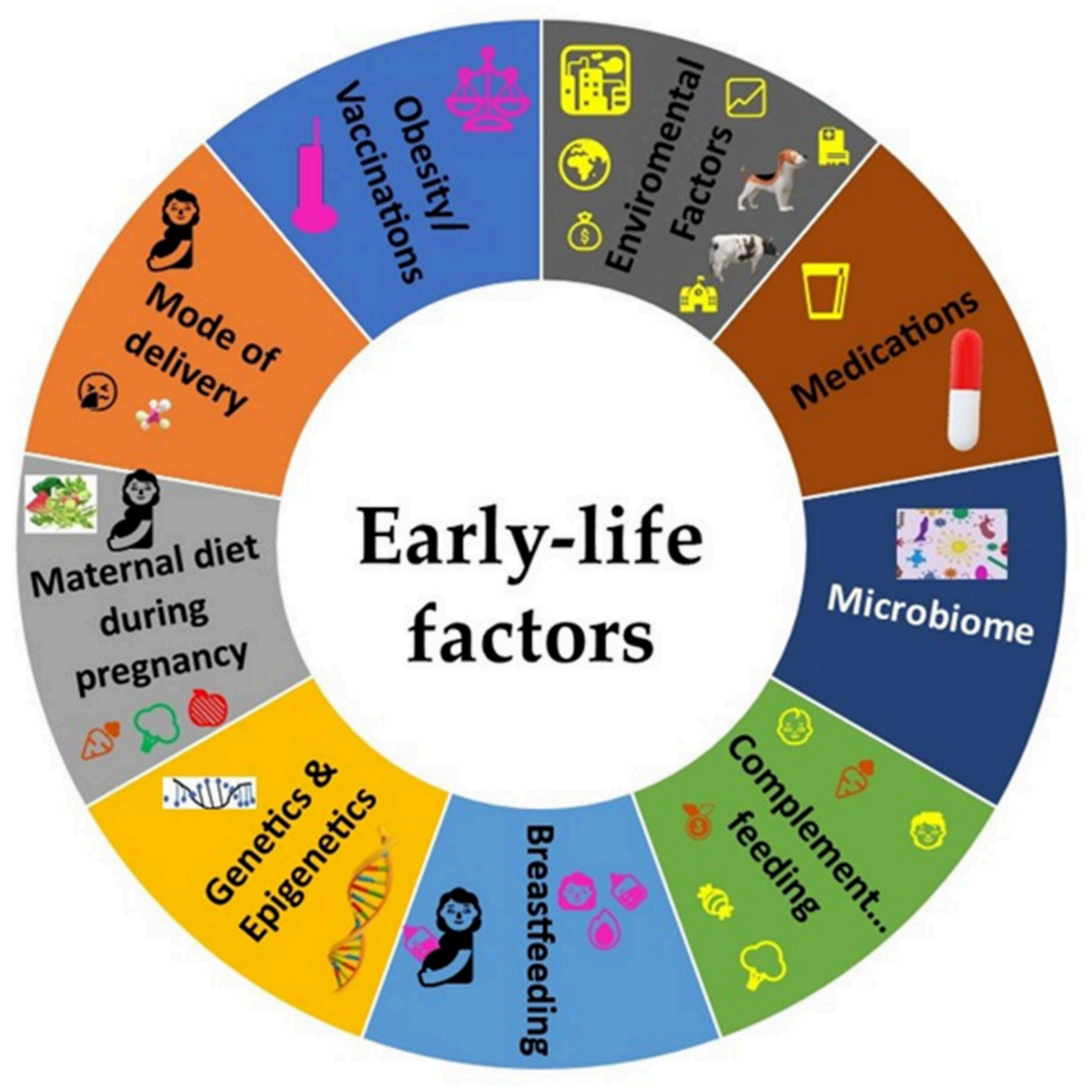
Early-life factors influencing health and immune development. Schematic overview of key early-life factors that may shape immune system development and subsequent health outcomes. These include maternal diet during pregnancy, mode of delivery, breastfeeding, complementary feeding, genetics and epigenetics, microbiome composition, medication exposure, environmental factors, and obesity and vaccination history. Collectively, these factors can interact during critical developmental windows to influence immune maturation and disease susceptibility later in life.

The available evidence therefore suggests that obesity may modify allergic susceptibility through interactions among inflammation, immune regulation, intestinal-barrier integrity, and the gut microbiome. However, human evidence remains more consistent for food sensitization than for clinically confirmed FA, while experimental studies support plausible biological mechanisms. Longitudinal studies are needed to determine whether obesity itself contributes to FA development or whether the observed associations reflect shared dietary, metabolic, and environmental factors [105,106,107,108,109]. Figure 2 can function as an integrative summary of all these early-life factors.

## 8. The Skin–Gut–Immune Axis

Atopic dermatitis (AD) is strongly associated with FA and represents an important component of the “atopic march”. Impaired skin-barrier integrity may facilitate cutaneous penetration of food allergens and promote allergen-specific IgE sensitization. In addition, inflammatory signals associated with AD may interact with the gut microbiota, intestinal function, and systemic immune responses, potentially influencing the development of oral tolerance [110,111].

The dual-allergen exposure hypothesis proposes that the route of allergen exposure may influence whether tolerance or sensitization develops. Observational and mechanistic evidence suggests that early gastrointestinal exposure to food allergens may promote immune tolerance, whereas exposure through inflamed or disrupted skin may favor sensitization. Environmental food allergens, including peanut proteins detected in household dust, may contribute to this process, while inhalational exposure has also been proposed as a potential route of sensitization [112,113].

The interaction between skin-barrier integrity, dietary exposure, and the gut microbiome may therefore be relevant to food-allergy development. Although interventions targeting the skin barrier and microbiome, including emollients, probiotics, and dietary approaches, have been investigated, clinical evidence that these strategies prevent FA remains insufficient [111].

AD should therefore be considered an important marker of FA susceptibility, particularly when barrier dysfunction is present. The available evidence supports a multifactorial model in which the route and timing of allergen exposure interact with epithelial integrity and immune development, providing a biological rationale for early oral allergen introduction while avoiding unnecessary dietary restriction [110,111,112,113].

## 9. Prevention: From Association to Intervention

Current prevention strategies increasingly emphasize modifiable early-life exposures rather than risk-factor identification alone. Among available interventions, randomized clinical evidence supports early introduction of allergenic foods—particularly peanut and egg—as the most consistently supported strategy for reducing the development of specific FA. Evidence for skin-barrier interventions, microbiome modulation, vitamin D supplementation, and other environmental strategies remains less conclusive [23,35,114,115,116,117,118,119,120].

In a large cluster-randomized trial, introduction of peanut, cow’s milk, wheat, and egg from 3 months of age reduced FA at 36 months compared with standard care (OR 0.40; 95% CI, 0.20–0.80). In contrast, a randomized trial of regular application of skin emollients did not significantly reduce food-allergy risk, indicating that emollient use alone is unlikely to provide primary prevention [33].

Current evidence therefore supports timely introduction of age-appropriate allergenic foods during complementary feeding, rather than deliberate avoidance or prolonged delay [23,114,116,118]. The optimal timing, quantity, frequency, and duration of exposure remain areas of ongoing research. In infants with severe atopic dermatitis or other features indicating increased risk, introduction of specific allergens may require individualized clinical assessment.

Other approaches—including skin-barrier protection, microbiome-directed interventions, vitamin D supplementation, maternal or neonatal nutritional interventions, and reduction in environmental exposures—remain under investigation. Although several have plausible biological mechanisms, clinical evidence remains insufficient to recommend these approaches specifically for the primary prevention of FA [35,115,117,119,120].

The prevention field is therefore shifting toward early, evidence-based intervention, with oral exposure to selected allergenic foods currently representing the most established strategy. Future research should clarify how nutritional, epithelial, microbial, genetic, and environmental factors interact and whether these factors can be integrated into individualized prevention approaches.

## 10. Knowledge Gaps and Future Directions

Despite substantial progress in understanding food-allergy development and prevention, important knowledge gaps remain. Much of the evidence linking early-life dietary, environmental, skin, and microbial exposures with FA is observational, limiting the ability to distinguish causal relationships from confounding. Greater understanding is needed of how genetic susceptibility, epithelial-barrier function, the gut microbiome, diet, and environmental exposures interact during critical periods of immune development [35,116,117,121].

Future studies should use standardized exposure assessment, clinically confirmed food-allergy outcomes, and long-term prospective follow-up, with greater representation of diverse populations and geographical settings. The optimal timing, dose, frequency, and duration of allergenic-food consumption also require further investigation [23,114,116,118]. Intervention studies should assess whether strategies targeting the skin barrier, microbiome, maternal and neonatal exposures, and other modifiable factors can prevent sensitization and clinically relevant FA. Although supported by biologically plausible mechanisms, these approaches currently lack sufficient evidence for routine use specifically for food-allergy prevention [35,117,119,120,121].

An important research direction is personalized prevention based on individual risk profiles. Integration of atopic dermatitis, genetic susceptibility, microbiome characteristics, and early-life dietary and environmental exposures may eventually allow preventive strategies to be tailored to high-risk infants. However, validated biomarkers and robust longitudinal and intervention evidence are still required before such approaches can be incorporated into clinical practice [35,119,121].

Future research should therefore move beyond observational associations and mechanistic hypotheses toward well-designed longitudinal and randomized studies that establish temporal relationships and, where possible, causality and translate mechanistic insights into effective, safe, equitable, and clinically applicable prevention strategies [23,35,114,116,117,118,119,120,121].

## 11. Conclusions

FA arises from a complex interplay between genetic susceptibility and early-life nutritional, microbial, epithelial, immune, and environmental factors. Pregnancy, infancy, and complementary feeding represent critical developmental windows during which these factors interact to shape immune maturation and oral tolerance. The gut microbiome, microbial metabolites, epithelial-barrier integrity, and the skin–gut–immune axis may contribute to this process, while genetic and epigenetic mechanisms may modify individual susceptibility. However, many reported associations remain observational and heterogeneous, limiting causal interpretation.

Among current preventive strategies, the most consistent evidence supports the timely introduction and regular consumption of allergenic foods, particularly peanut and egg, during complementary feeding, rather than prolonged allergen avoidance. Conversely, maternal dietary restriction, breastfeeding as an independent preventive intervention, vitamin D or omega-3 supplementation, hydrolyzed formulas, probiotics, microbiome-directed interventions, and environmental modifications cannot currently be recommended specifically for food-allergy prevention in the general population.

FA prevention should therefore move beyond a single-factor model toward an integrated understanding of how nutrition, microbiota, epithelial barriers, genetic susceptibility, and environmental exposures interact during early immune development. Future longitudinal and randomized studies integrating dietary, genomic, epigenomic, microbiome, metabolomic, immune, and environmental data with clinically confirmed outcomes may clarify causal pathways and identify modifiable targets. Such approaches could ultimately support risk-stratified, personalized, and evidence-based strategies for food-allergy prevention from the earliest stages of life.

## Figures and Tables

**Table 1:** Early-life factors associated with food-allergy risk.

Factor	Proposed Effect/Association with FA	Potential Mechanisms	Strength/Limitations of Evidence
Maternal diet	Mediterranean diet: possible modest protective association; evidence inconsistent	Microbiome, metabolites, immune development	Mostly observational
Breastfeeding	No consistent evidence of independent prevention	Immune factors, microbial colonization	Confounding and heterogeneity
Timing of allergenic-food introduction	Early introduction, particularly peanut and egg, reduces risk of specific FA	Oral tolerance, Treg development	Strongest intervention evidence
Dietary diversity	Greater diversity may be associated with lower allergic outcomes	Microbiome maturation, immune education	Mainly observational
Antibiotic exposure	Associated with increased allergy/FA risk in some studies	Microbiome disruption, immune modulation	Observational; confounding
Gut microbiome	Dysbiosis associated with FA phenotypes	Microbial metabolites, epithelial and immune regulation	Heterogeneous; no consistent signature
SCFAs	Higher early-life SCFAs generally associated with lower allergic outcomes	GPCR signaling, HDAC inhibition, Treg regulation	Associations heterogeneous
Intestinal barrier	Barrier disruption may increase sensitization	Increased antigen penetration, epithelial signaling	Mechanistically plausible
Atopic dermatitis	Strongly associated with FA	Skin-barrier dysfunction, epicutaneous sensitization	Strong association
Air pollution	PM_2.5_ associated with increased odds of childhood FA	Oxidative stress, epithelial and immune effects	Mostly observational
Farm/pet/sibling exposure	Possible protective associations	Microbial diversity and immune maturation	Stronger for atopy than confirmed FA
Daycare	Inconsistent association	Microbial exposure, infections	Conflicting evidence
Obesity	More consistently associated with food sensitization than confirmed FA	Inflammation, barrier dysfunction, microbiome	Limited human evidence for clinical FA
Vaccination	No evidence of increased FA risk		

## Data Availability

No new data were created or analyzed in this study. Data sharing is not applicable to this article.

## Abbreviations

The following abbreviations are used in this manuscript:

FA	Food allergy
RORγt	Retinoic acid receptor-related orphan receptor gamma t
Treg	T regulatory
SCFAs	Short-chain fatty acids
TLRs	Toll-like receptors
EAACI	European Academy of Allergy and Clinical Immunology
ASM	Acid-suppressive medications
PPIs:	Proton-pump inhibitors
PPAR-γ	Peroxisome proliferator-activated receptor gamma
FLG	Filaggrin
SERPINB7	Serpin family B member 7
HLA	Human Leukocyte Antigen
IL	Interleukin
DNA	Deoxyribonucleic Acid
AD	Atopic dermatitis
